# Effect of Duration of High-Grain Feeding on Chewing, Feeding Behavior, and Salivary Composition in Cows with or without a Phytogenic Feed Supplement

**DOI:** 10.3390/ani12152001

**Published:** 2022-08-08

**Authors:** Raul Rivera-Chacon, Sara Ricci, Renée M. Petri, Andreas Haselmann, Nicole Reisinger, Qendrim Zebeli, Ezequias Castillo-Lopez

**Affiliations:** 1 Institute of Animal Nutrition and Functional Plant Compounds, Department for Farm Animals and Veterinary Public Health, University of Veterinary Medicine Vienna, Veterinärplatz 1, 1210 Vienna, Austria; 2 Christian Doppler Laboratory for Innovative Gut Health Concepts of Livestock, Veterinärplatz 1, 1210 Vienna, Austria; 3 Division of Livestock Sciences, Department of Sustainable Agricultural Systems, BOKU-University of Natural Resources and Life Sciences, Gregor-Mendel-Straße 33, 1180 Vienna, Austria; 4 BIOMIN Research Center, BIOMIN Holding GmbH, 3430 Tulln, Austria

**Keywords:** dairy cow, rumination, saliva, feed sorting, phytogenic feed additive

## Abstract

**Simple Summary:**

This study evaluated the effects of the duration of high-grain feeding and a phytogenic feed supplement on the chewing, eating, and lying behavior as well as the salivation dynamics in dairy cows. A control group of cows with no supplementation was compared to a group receiving a phytogenic feed supplement. An increased duration of the high-grain diet increased meal size, but reduced rumination, the total chewing time, and the chewing index. Similarly, as the experiment progressed, the cows sorted against short feed particles. The results also showed that the duration on the high-grain diet increased the salivary pH; however, the salivary phosphate decreased at the start of high-grain feeding. Feed ensalivation also decreased after 4 weeks of consuming the high-grain diet. The supplemented cows sorted in favor of fiber-rich feed particles in week two and had greater salivary pH in week four on the high-grain diet. Our study showed that the duration of feeding exacerbates the negative impacts of high-grain diets in cows. However, supplementation with the feed additive mitigated some of these negative effects.

**Abstract:**

Switching diets from forage to a high-grain (HG) diet increases the risk of rumen fermentation disorders in cattle. However, the effects of the duration of the HG feeding, after the diet switch, on animal behavior and health have received considerably less attention. This experiment primarily aimed to assess the effects of the duration of an HG diet on the chewing, eating, and lying behavior and salivation dynamics in a control group (CON) and a group of cows receiving a phytogenic feed supplement (TRT) at 0.04% (DM basis), which included L-menthol, thymol, eugenol, mint oil, and cloves powder. The experiment was a crossover design with nine non-lactating cows, and two experimental periods with an intermediate washout of four weeks. In each period, the cows were first fed a forage diet for a week to collect baseline measurements representing week 0; then, the diet was switched over a week to HG (65% concentrate), which was fed for four continuous weeks (week 1, week 2, week 3, and week 4 on an HG diet, respectively). The cows were divided in two groups of four and five animals and were randomly allocated to CON or TRT. The data analysis revealed that at the start of the HG feeding, the dry matter intake and the cows’ number of lying bouts increased, but the eating time, rumination time, and meal frequency decreased, resulting in a greater eating rate. We also found that an advanced duration on an HG diet further decreased the rumination time, total chewing time, chewing index, and sorting in favor of short feed particles, with the lowest values in week 4. The feed bolus size increased but feed the ensalivation decreased in week 4 compared to week 0. The dietary switch increased salivary lysozyme activity, and the advanced duration on the HG diet increased salivary pH, but salivary phosphate decreased in weeks 1 and 2 on the HG diet. Supplementation with TRT increased sorting in favor of physically effective NDF (peNDF) in week 2 and increased salivary pH in week 4 on an HG diet. Overall, the negative effects of the HG diet in cattle are more pronounced during the initial stage of the HG feeding. However, several detrimental effects were exacerbated with the cows’ advanced duration on feed, with host adaptive changes still observed after 3 and 4 weeks following the diet switch. The TRT mitigated some of the negative effects through the temporal improvement of the salivary properties and the intake of peNDF, which are known to modulate rumen fermentation.

## 1. Introduction

Feeding cattle high-grain (HG) diets is commonly implemented in the dairy and beef cattle production industry to enhance the energy intake for milk or accelerate daily gains, respectively. Grains are rich in starch and less voluminous and are thereby a better source of metabolizable energy than forages for cattle diets in many parts of the world. However, feeding large amounts of grain is known to influence chewing behavior, which could affect animal health due to the increased risk of gut disorders. More specifically, HG diets have been reported to impair rumination and total chewing times, which are essential physiologic processes in ruminants [1,2,3,4] often used as indicators of cattle welfare and health [5]. Additionally, the lying behavior of cattle is an important indicator of their comfort and welfare [6]. For instance, Haley et al. [7] demonstrated that lying time is closely related to comfort and health changes. Cattle ruminate more while lying down than when standing. However, feeding them HG diets may change the lying behavior of cows [2], with longer lying times and shorter rumination times usually reflecting distress and discomfort [8].

The feed-sorting behavior in cattle may also be affected by a change in dietary composition [9,10,11]. For example, cows are known to select diet fractions during eating, sorting for shorter particles in the ration (concentrate) and refusing longer particles (forages) [12,13,14]. Another report by Greter and DeVries [15] demonstrated that cows fed with a 54% grain diet sorted against long particles and tended to sort in favor of short particles. Nonetheless, this feeding behavior may contribute to further impaired chewing activity and salivation due to the reduced intake of dietary physically effective NDF (peNDF) and could consequently affect animal health or gut function. Specifically, peNDF in the diet is important because it stimulates chewing activity, greater salivary buffer secretion, and the regulation of ruminal pH [16]. Therefore, diet composition plays an important role in feed sorting, with the time invested in eating and ruminating positively correlated with the intake of peNDF [17]. In this regard, diets with more fiber are associated with more meals per day and reduced eating rates [18], which positively modulates rumen fermentation.

The essential physiologic role of chewing in cattle is based on its contribution to stimulating salivary secretion [19,20]. Salivary buffers help stabilize the ruminal pH [21] because salivary buffers such as bicarbonate and phosphate represent important components for ruminal proton removal in the rumen [22]. Furthermore, salivary secretions contain different proteins such as mucins, lysozymes, and immunoglobulins [23], which contribute to health and gut function [24]. Therefore, an increase in mastication and salivary flow can enhance the rumen acid–base balance and ultimately improve health [5,25]. In this context, phytogenic compounds, such as thymol and thyme oil, have shown a potential to modulate the salivary secretions in cattle. Similarly, menthol has been reported to stimulate chewing and increase salivation in non-ruminants [26,27].

The dietary shift to HG feeds is known as the time with the greatest risk for cattle health due to the major adaptive changes occurring in the host during this interval. However, there is limited research on the effects of the duration of the HG feeding challenge after the dietary change on salivary secretions [28] as well as the chewing activity and lying behavior. In addition, there is a paucity of information regarding the effect of the supplementation of feed with phytogenic compounds on salivary composition and production [29], chewing activity, or the eating behavior of cows. Thus, there is a need to fill or strengthen these research gaps in the scientific literature. Therefore, the aims of this study were to evaluate the effect of the duration of an HG feeding challenge on the chewing activity, eating and lying behavior, and salivary composition and production in dairy cows without or with phytogenic feed supplementation. Our hypothesis stated that the advanced duration of the HG challenge would exacerbate the negative effects on rumination, the eating and lying behavior, and the salivary production and composition. We also hypothesized that the phytogenic supplementation would alleviate the decrease in the chewing activity as well as improve the feed sorting and salivary properties.

## 2. Materials and Methods

### 2.1. Animals, Experimental Design, Treatments and Management

This study was part of a larger experiment; details on animal management, feeding, as well as the results regarding ruminal fermentation have been published in a companion paper [30]. The animal protocol was approved by the institutional ethics and animal welfare committee of University of Veterinary Medicine Vienna and the Austrian national authority (protocol number: BMBWF- 68.205/0003-V/3b/2019).

**Figure 1:**
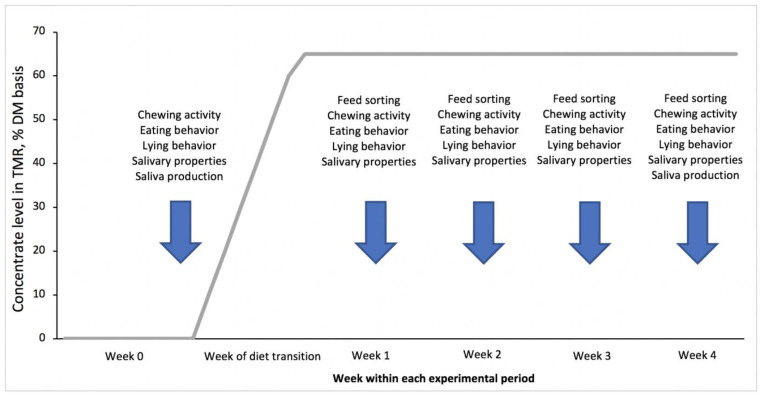
General outline of each of the 2 experimental periods illustrating the dietary adaptation to high-grain (HG) feeding and the duration of the HG feeding challenge. The blue arrows indicate the time points for major samplings as well as measurements performed.

Briefly, we used nine rumen-cannulated, individually-fed, dry Holstein cows in a cross-over experimental design. Cows were blocked in 2 groups of 4 and 5 animals and assigned to a control diet (CON) or a diet with 0.04% (DM basis) of a phytogenic feed additive based on a blend of mint oil (*Mentha arvensis*), cloves powder (*Syzygium aromaticum*) and thymol, including L-menthol and eugenol (TRT; Digestarom^®^, DSM GmbH). The inclusion rate of the phytogenic supplement was defined based on previous studies. Details of this product have also been reported in the companion paper [30]. Each experimental period consisted of 6 weeks. During the first week of each period, cows were fed a solely forage diet including 45% grass silage, 45% corn silage, and 10% grass hay (DM basis). This week of forage feeding was used to collect baseline measurements representing week 0. In the following week, an HG feeding challenge was induced through an increment in the proportion of concentrate in the total mixed rations (TMR, 10% daily increments, DM basis). After the dietary change, the HG ration contained 26.25% grass silage, 8.75% corn silage, and 65% of a pelleted concentrate based on barley and triticale ground grains (DM basis; Supplementary Table S1), and this HG ration was fed for 4 consecutive weeks (week 1, week 2, week 3, and week 4 on the HG, respectively). Figure 1 depicts the overall experimental outline for each of the 2 periods. Between these experimental periods there was a washout interval, which lasted 4 weeks.

During the experiment, cows were housed in a free-stall barn with deep litter cubicles (2.6 × 1.25 m, straw litter) and free-choice mineral blocks. Before the initiation of the study, cows were randomly allocated to the feed bins, so that each cow was trained using an ear tag transponder to allow access to only one feed bin throughout the experiment. Therefore, individual feed intake was continuously recorded since each feed bin was equipped with an electronic scale (Insentec B.V., Marknesse, The Netherlands). This feeding approach enabled the collection of data related to eating behavior and feed sorting for each cow. Additionally, there was no feed bunk competition due to space limitations or determined by the social hierarchy because each cow had her own feed bin. Therefore, each cow was used as an experimental unit. Diets were automatically mixed every day (Trioliet Triomatic T15, Oldenzaal, The Netherlands), and were offered in the individual feed bins to each cow at 0800 h. When needed, the amount of water added to the diet was adjusted during mixing to target 46% DM in the TMR. Cows were fed targeting 10% of feed refusals.

### 2.2. Collection of Feed Samples and Analyses for Chemical Components

Dry matter concentration of the TMR was determined every day by drying samples at 100 °C for 24 h. Using these data, DM content of the TMR was adjusted if needed by changing the amount of water added to the diet during mixing. Individual feed samples were collected at the beginning and at the end of each experimental period, while TMR samples were collected once a week for chemical composition. Chemical composition of each TMR sample was evaluated, and then values were averaged by chemical component across sampling weeks. Details on the laboratory analyses as well as the method used for the evaluation of particle size distribution of the rations have been reported in the companion paper [30].

### 2.3. Evaluation of Chewing, Feed Sorting and Eating Behavior of Cows

Evaluation of chewing activity was conducted weekly (Figure 1), with the first evaluation performed in week 0, and the following conducted in each of the 4 weeks of the HG feeding regimen. This analysis included eating time, ruminating time, number of chews per minute and per feed bolus, chewing index, drinking time, and drinking gulps. These parameters were evaluated following Kröger et al. [31]. To do so, noseband pressure sensors (RumiWatch System, ITIN + HOCH GmbH, Fütterungstechnik, Liestal, Switzerland) were used for 3 consecutive full-days each week in all cows simultaneously. The evaluation of chewing activity with these systems is based on the detection of changes in pressure, which are monitored by the sensors when cows ruminate or eat, and according to the animal’s head position. Halters were placed on the cows for adaptation 12 h before the initiation of data collection for chewing activity. After measurements were completed, the recorded raw data were transferred using the interface software RumiWatch Manager (version 2.2.0.0; Itin and Hoch GmbH) and processed with the evaluation software RumiWatch Converter (Version 0.7.3.2). Graphs outlining diurnal variations of rumination and eating time were constructed for a detailed description of a full-day period within each experimental week.

Feed sorting behavior was evaluated for each cow once a week (Figure 1), starting from the first week of the HG feeding regimen using the methodology described by Haselmann et al. [32] and Stauder et al. [3]. Specifically, particle size distribution of TMR offered and in the feed refusals collected in the following day were measured. Samples of TMR were collected; on the following day, refusal samples were collected from each feed bin in the morning before the new feed was offered. Feed sorting for each cow was expressed through the change in particle size distribution (as-fed basis) of the provided TMR in relation to the refusals. According to Leonardi and Armentano [12], feed selection of each particle size was calculated as the percentage of the actual as-fed intake from the predicted as-fed intake, expressed as the selection index. Predicted intake of a specific particle size was estimated as the product of as-fed intake and the proportion of this specific fraction in the offered TMR.

Eating behavior was evaluated for each cow weekly (Figure 1). The variables evaluated included valid visits, visit duration, visit size, meal frequency, meal duration, meal size, and eating rate. A valid visit was defined when a cow stayed at the feeder for 4.5 min and consumed at least 200 g of DM. Additionally, when the time interval between the end of one visit and the start of the next one was shorter than 29.5 min, these visits were considered as part of a single meal. This time interval was calculated based on the methods described by Tolkamp et al. [33] and DeVries [34]. Eating rate was estimated following the protocol of Beauchemin et al. [35].

### 2.4. Evaluation of Lying Behavior

Lying behavior was measured in one of the 2 experimental periods. These measurements were collected during the same 3 days used for evaluation of chewing activity and was performed in all cows using data loggers (HOBO Pendant G Acceleration Data Logger). For this purpose, loggers were placed on the external side of the hind left leg using a self-adherent bandage (UKAL cohesive flexible bandage, France). Prior to the attachment, each logger was fixed to a silicon mat to avoid chafing. The recording interval was set at 30 s. After 3 days of data collection, the loggers were removed, and raw data were downloaded using the software HOBOware PRO. Lying data were processed using the Ledgerwood’s algorithms for lying/standing bouts and laterality [36]. Lying behavior variables were calculated on a daily basis and included: standing time, total lying time, lying time on the left side, lying time on the right side, total lying bouts, lying bouts to the left, and lying bouts to the right.

In addition, the data of rumination activity collected in the corresponding experimental period were combined with the lying behavior. To perform these calculations, both parameters were matched in 10 min intervals. This enabled the calculation of rumination times while standing or lying. For these calculations, we considered a minimum time of 9.5 min to assume that cows were lying for the complete 10-min interval.

### 2.5. Saliva Collection and Evaluation of Salivary Characteristics, and Measurement of Saliva Production

Saliva samples were collected orally once a week (Figure 1) to evaluate salivary physico-chemical characteristics, with the first collection conducted in week 0, and the following collections performed in each of the 4 weeks of the HG feeding regimen. Details of saliva samplings have been described by Castillo-Lopez et al. [28] and Ricci et al. [37]. Briefly, saliva collections were conducted with a vacuum pump before the morning feeding. Then, aliquot samples were frozen immediately at −20 °C. At the end of the experiment, samples were thawed, and pH was measured using a portable pH meter (Mettler-Toledo, AG; Analytical CH; Schwerzenbach, Switzerland). Additionally, other salivary physico-chemical characteristics including buffer capacity, bicarbonate, phosphate, mucins, lysozyme activity, and total proteins were measured at the end of the experiment as previously described by Castillo-Lopez et al. [28].

The measurement of salivary production and evaluation of feed boli characteristics were conducted twice during each experimental period, in week 0 and week 4. The protocols for collection of feed boli and sample analyses as well as calculations are also reported in detail in Castillo-Lopez et al. [28]. The evaluated parameters related to salivary production included feed boli size, saliva content in feed boli, salivation rate (g saliva flow/min), and feed ensalivation (g saliva/g feed DM).

### 2.6. Statistical Analysis

Statistical analyses were performed using the Proc Mixed of SAS (version 9.4; SAS Institute, Cary, NC, USA). Data were checked for outliers and normality, if the normality assumption was not met, transformations were performed as described in Rivera-Chacon et al. [30]. The statistical model included the fixed effects of the experimental period, duration of the HG feeding regimen in weeks, TRT supplementation, and the interaction between duration of the HG feeding regimen × phytogenic supplementation. The cow was included as random effect in the model. In addition, data from the same cow at different times were analyzed as repeated measures, with a first order variance-covariance structure matrix. Each cow was considered as the experimental unit. The multiple comparisons of means were performed using the PDIFF option. The transformed data were back-transformed after the analysis of variance. Additionally, we conducted Pearson correlation analyses using Proc corr of SAS between rumen fermentation variables (ruminal pH and short chain fatty acids) vs. rumination time, total chewing time, salivary properties (bicarbonate content, phosphate content, buffer capacity, and pH), feed insalivation, and feed sorting. From a preliminary statistical power analysis that we conducted [29] according to Stroup [38] and Kononoff and Hanford [39], using similar variables as those evaluated in this study, we observed that a minimum of n = 4 is required to obtain a statistical power of 0.82 with α = 0.05, an acceptable level.

We report the results as LSM as well as the largest standard error of the mean (SEM). Statistical significance was declared when *p* ≤ 0.05, and statistical tendencies are discussed if 0.05 < *p* ≤ 0.10.

## 3. Results

### 3.1. Dietary Characteristics

During the week of the diet change, the dietary composition drastically shifted from 50.4 to 30.9% NDF. Additionally, there was also an abrupt reduction in fibrous long feed particles and peNDF. At the same time, there was an increase in the dietary starch content (Supplementary Table S1). Thus, this diet shift represented an adequate experimental approach to induce an HG feeding challenge on the animals and to evaluate their host adaptive responses with respect to advanced days of feeding.

### 3.2. Chewing Activity and Eating Behavior

The rumination time was strongly impaired by the duration of the HG feeding and decreased from 348 to 245 min/day from week 1 to week 4 of the HG feeding regimen, independent of the TRT. Similarly, the total chewing time (minutes of chewing per day) was highly influenced by the duration of the HG feeding regimen with a pronounced reduction observed in week 4 on an HG diet (*p* < 0.05). The chewing index (total chewing time/kg DMI) was markedly decreased at the start of the high-grain challenge, and the values were maintained at low levels throughout the entire HG feeding period. In addition, the eating time showed a decreasing pattern with the lowest value found in week 4 of the HG feeding (145 min per day), independent of the TRT. Nonetheless, drinking gulps and drinking time followed a different pattern, which increased as the HG diet was implemented (*p* < 0.05) and both variables reached the highest values in week 4 on the HG (Table 1).

The dry matter intake (DMI) was greater for week 1 on an HG diet compared to week 0, with estimates of 8.0 and 13.0 kg for week 0 and week 1 on the HG, respectively. The greatest DMI was reached in week 3 on the HG, with an average of 14.1 kg. Furthermore, eating and feed bunk visits were mostly affected immediately at the start of the HG challenge. For instance, the visit duration (min) and meal frequency (#/day) decreased, whereas the visit size (kg DMI per visit) increased from week 0 to week 1 of the HG feeding regimen. In addition, the meal size (kg of DM) and eating rate increased immediately from the start of the HG feeding. The advanced duration on the HG diet increased the meal size compared to week 1 on the HG. We also found that supplementation with TRT tended to reduce the meal size in week 2 of the HG feeding regimen (Table 2).

**Figure 2:**
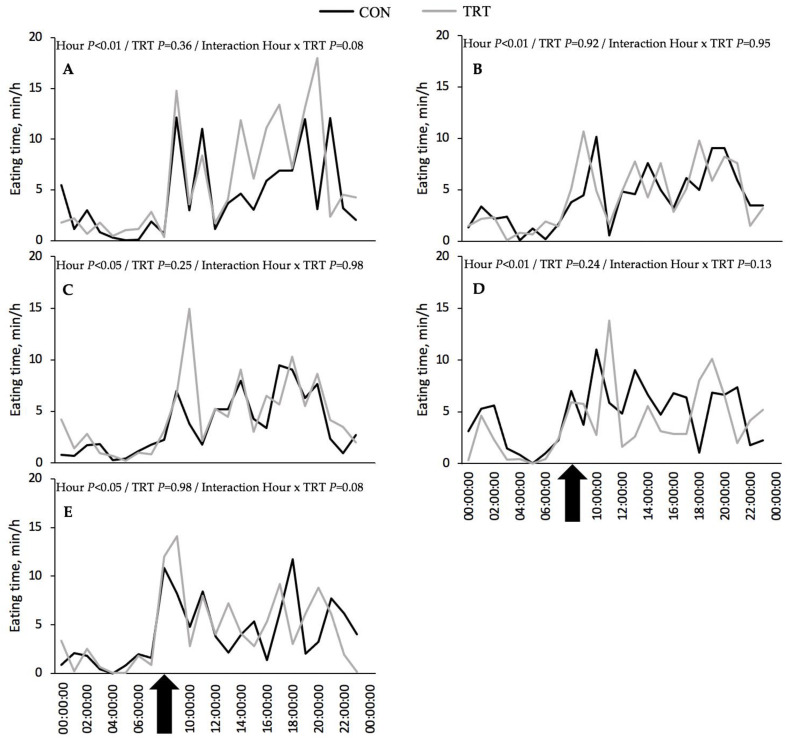
Diurnal variation of eating time according to duration on a high-grain diet in a control group of Holstein cows (CON) or a group receiving a phytogenic feed supplement (TRT) based on L-menthol, thymol, eugenol, mint oil, and cloves powder (0.04%, DM basis). (**A**)—Week 0; (**B**)—Week 1 on high-grain; (**C**)—Week 2 on high-grain; (**D**)—Week 3 on high-grain; (**E**)—Week 4 on high-grain. The black arrows indicate the time of feeding.

From week 1 of the HG feeding regimen, there was a change in the pattern of the time spent eating throughout the day. Specifically, in week 0 (Figure 2A), there were multiple peaks for eating time distributed during the first 13 h after feed delivery. However, from week 1 of the HG feeding regimen onwards (Figure 2B–E), the eating time decreased, and the predominant peak of the eating time was generally observed shortly the after delivery of the diet in the feed bins early in the morning.

### 3.3. Feed Sorting Behavior

The feed-sorting behavior analysis showed that in week 3 on the HG diet, sorting for medium size feed particles decreased (*p* = 0.01) independent of the TRT. In addition, sorting for short size feed particles tended (*p* = 0.08) to decrease due to the advanced duration of the HG diet. With regard to feed supplementation, during week 2 of the HG feeding regimen, the TRT group sorted in favor of larger particles of feed, while the CON cows followed the opposite pattern. However, in weeks 3 and 4 on the HG diet, both groups sorted in favor of larger feed particles. In addition, in week 2 on the HG diet, TRT cows showed a greater preference for peNDF than CON (*p* < 0.05; Table 3).

### 3.4. Lying Behavior and Rumination According to Animal Position

There was a tendency for the total standing time to decrease at the start of the HG feeding (*p* = 0.10), and the total lying time tended to increase (*p* = 0.10), but with no effect from the TRT supplementation (*p* = 0.93). The total lying bouts and lying bouts to the left or right side were greater from week 1 on the HG diet onwards compared to week 0, with these variables being greater (*p* < 0.05) for TRT in week 2 of the HG diet compared to the CON cows. There was a tendency for a greater time spent lying on the left side for the TRT compared with CON cows (*p* = 0.10) in week 1 of the HG diet (Table 4).

The rumination time while standing and while lying on the right or left decreased consistently (by 56, 63, and 60%, respectively) from week 0 and throughout the 4 weeks on the HG diet (*p* < 0.05; Table 4). In general, rumination times while lying down on the left side were greater than rumination time on the right side. In addition, the supplementation increased the rumination time while lying on the left side in week 1 on the HG diet (*p* < 0.05) compared to the CON.

### 3.5. Feed Bolus Ensalivation and Salivary Physico-Chemical Properties

Feed bolus size (as-is or on a DM basis) was greater in week 4 of the HG feeding challenge (*p* < 0.05) compared to week 0. The total amount of saliva in the feed bolus and the flow of saliva did not show a diet effect (*p* = 0.97), but the feed ensalivation (g saliva/g feed bolus) was highly influenced by the diet, with a strong reduction of 51% observed when the HG diet was fed (*p* < 0.01; Table 5). Supplementation with TRT did not change the feed bolus or saliva flow (*p* ≥ 0.13). The Pearson correlation analyses showed a positive correlation between the ruminal pH and feed ensalivation (r = 0.54; *p* < 0.01), a negative correlation between the ruminal total short chain fatty acids and feed ensalivation (r = −0.53; *p* < 0.01), and a positive correlation between the ruminal acetate to propionate ratio and feed ensalivation (r = 0.62; *p* < 0.01).

Several salivary physico-chemical properties changed due to the diet shift or the duration of the HG feeding regimen (Table 6). For example, we observed an increase in salivary pH with the increased duration on the HG diet, with the greatest values observed in weeks 3 and 4 on the HG diet. Compared to the CON, the TRT supplementation increased salivary pH (*p* < 0.05) in week 4 on the HG diet. In addition, the salivary lysozyme activity increased from the start of the HG feeding (*p* < 0.05), reaching the highest values in weeks 3 and 4 on the HG diet, an average increase of around 45% compared to week 0. On the other hand, we found a reduction in the salivary phosphate concentration during weeks 1 and 2 of the HG feeding regimen, but this variable increased in weeks 3 and 4 on the HG diet. In addition, TRT supplementation tended to increase the salivary buffer capacity (*p* = 0.09) in week 3 on the HG diet compared to the CON.

The concentrations of salivary bicarbonate, total mucins, and total proteins were not affected by the change in diet or the duration of the HG feeding. However, considering the reduction in feed bolus ensalivation, there was an overall reduction in the supply of these salivary components per gram of feed bolus.

## 4. Discussion

This study aimed to evaluate the effect of the duration of an HG feeding challenge on chewing, eating, and lying behaviors as well as the salivary production and composition in cows with or without a phytogenic feed supplement. In agreement with our hypothesis, there was a reduction in the rumination time not only at the start of the HG feeding regimen, but also due to advanced duration on the HG diet. Consequently, the reduction in the rumination time likely contributed to the lower feed ensalivation observed after 4 weeks of the HG feeding regimen. Furthermore, the total chewing time decreased because of the duration on the HG diet. Chewing activity is essential for adequate rumen function because it stimulates salivation and normal rumen fermentation [16]. It has also been reported that a greater chewing time improves feed digestion by exposing nutrients and increasing feed surface area, thereby facilitating the activity of microbial enzymes [41]. Therefore, our findings clearly indicate that the duration on an HG diet exacerbates the negative effects on the chewing activity in cattle. Our observations for rumination time were lower than the values reported by Ben Meir et al. [42] for lactating cows consuming diets with similar levels of concentrate. These contrasting findings may be because of the higher DMI intake in lactating cows used by Ben Meir et al. [42], with intakes twice as high as the dry cows utilized in our study, resulting in more ruminal digesta available for rumination. Although there are limited data regarding the effects of phytogenic supplementation on rumination, the research conducted by our group showed that a blend of essential oils increased the rumination time during the first 2 weeks of an HG feeding challenge compared to a control TMR [43]. In another experiment, Castillo-Lopez et al. [29] demonstrated in a short-term trial that thymol supplementation tended to increase chews/min. However, in the present study, this effect was not observed, suggesting that thymol may exert only a temporary stimulating effect on chewing activity.

Our findings also indicate that the DMI increased from the first week of the HG feeding regimen. These results support reports from Dann et al. [44] for diets containing similar levels of starch. The increase in the DMI might have occurred because of the small particle size of the HG diet, which allows for a greater feed intake due to the decreased gut fill. In addition, the greater feed consumption may be explained by the improved feed acceptability with the inclusion of concentrate in the diets. Our findings showed a greater meal size and eating rate due to the advanced duration on the HG diet, which is a factor that increases the risk of ruminal acidosis, because this results in the accumulation of volatile fatty acids in the rumen. The latter observations explain the greater feed boli measured after 4 weeks on the HG. In general, the number of meals per day observed in this study were lower compared to previous studies in lactating cows [18,33,42]. This may be because the nutrient utilization and energy metabolism are slower in dry cows compared to lactating animals. With regard to the effect of the TRT on the DMI, reports suggest that individual phytogenic compounds may influence feed intake [29]. In this trial, the TRT tended to reduce meal sizes in week 2 on the HG diet compared to the CON. This may be explained by the increased intake of long feed particles and peNDF for the TRT in that week, which contributed to gut fill. The increased preference for long feed particles possibly was due to olfactory and gustatory stimulation of TRT. This is a beneficial effect because of the role of fibrous feed ingredients in the regulation of ruminal pH, particularly when there is a need to modulate fermentation due to a low ruminal pH, as reported in the companion paper [30]. Furthermore, the findings from the correlation analyses in this study agree with our expectations and showed that a greater amount of saliva per gram of the feed that cows consume contributes to an increased ruminal pH by neutralizing the acids produced in the rumen. The positive association between the feed ensalivation and the ratio of acetate to propionate agrees with the simultaneous change in the feed ensalivation and acetate production due to a change in the proportion of the concentrate in the diets. For example, forage-based rations are associated with greater feed ensalivation and an increased acetate production.

The majority of the studies on feed sorting behavior have evaluated feed management or the fiber of the diet [3,25,45], but the influence of the duration of the HG challenge or phytogenic supplementation on feed sorting remains yet to be elucidated. Interestingly, the present study showed that the advanced duration on the HG diet decreased the cows’ preference for the short feed particles. This may reflect a response of the cows to counteract the negative effects of low fiber diets on rumen pH, because short feed particles are rich in readily fermentable starch that increase ruminal fermentation and acidification.

Our hypothesis also stated that the lying time would increase with the duration of the HG feeding regimen. Several experiments have demonstrated that there is a close relationship between the standing and lying time and laminitis in cows. For example, reduced lying times and abnormal standing times seem to be indicators of the development of laminitis [46]. In this study, we found that there was an increase in the number of lying bouts on either the right or left side from the start of the HG feeding regimen, and these increased values were maintained throughout the HG feeding challenge. Greater lying bouts may reflect animal discomfort and may be due to the effect of the acidogenic diets that lead to damage in the lamina of the foot [47]. Furthermore, Fukasawa et al. [48] reported similar results, describing a tendency to increase lying bouts when implementing high concentrate feeding compared to forage feeding. The higher nutritive value of the diet has been suggested to influence lying time or lying bouts as well, with increasing lying times associated with a greater body condition score [6]. In this study, there was an increase in the body weight of cows (69 kg), which could have contributed to the greater lying bouts throughout the HG feeding.

Our results show that laterality of the lying behavior followed a similar pattern as reports from Tucker et al. [49], with a fairly even distribution between the left or right lying times. Previous research has demonstrated that cows preferably ruminate while lying down [17,50], which coincides with our findings. It is possible that as the cows experienced increased discomfort as a consequence of intensive rumen fermentation, they preferably ruminated while lying on the left side. These results are supported by pioneering findings by Bailey and Balch [51] and Albright [52], who suggested that lying on the left side is a strategic position for cows to increase rumination efficiency, because this position may facilitate the regurgitation process of the digesta due to the improved alignment of the esophagus with the rumen contents. In addition, the TRT group increased its propensity to lie on the left compared to CON, especially in the second week on the HG diet. Nonetheless, the exact association between the TRT supplementation and the lying side of cows remains to be elucidated.

Another aim of this study was to evaluate the changes in salivary composition and dynamics. In agreement with our hypothesis and with previous studies [28,35], the change to an HG diet had a negative impact on feed ensalivation. Beauchemin et al. [35] also suggested that eating time may influence salivary secretion in cows, which supports our findings showing that a lower eating time was associated with a lower feed ensalivation in week 4 on the HG diet. Moreover, salivary secretion is not influenced by rumination alone. For instance, it has been demonstrated that when the eating rate increases, there is a reduction in the daily salivation of cows [35]. This supports our findings showing that when the cows consumed the HG rations at a faster pace, the feed ensalivation was lower. With regard to the effect of phytogenic compounds on salivation, the increased salivation rate via individual phytogenic compounds previously reported [37] in a short-term trial was not confirmed in the present study. These contrasting findings may be because the stimulus for the salivation flow of individual phytogenic compounds could decrease when combined with other compounds. It is also possible that differences in the effects of these compounds are related to their distinct modes of action, with some substances being active in the oral cavity [53], while others influence salivary secretion through the olfactory stimulation of the nervous system [54,55].

Salivary physico-chemical properties play important roles in gut function and health. In this trial, we observed that salivary lysozyme activity increased at the start of the HG feeding regimen. The salivary lysozyme is known to act as an antimicrobial bioactive component [56,57]. Thus, this observation may reflect a host response to counteract a potential outgrowth of pathogens due to the drastic diet shift [57]. Additionally, we found that salivary pH increased with the advanced time on the HG diet, which may be a host response for ruminal pH regulation, given the role of saliva for proton removal and ruminal pH balance. On the other hand, we found a reduction in salivary phosphate during the first 2 weeks on the HG, an observation that is highly relevant because of the role of this salivary buffer in the regulation of ruminal pH. The latter effect may exacerbate the reduction in ruminal pH commonly observed when cattle are switched from forage to HG rations. Another finding was the tendency for TRT to increase the salivary buffer capacity in the third week of the HG feeding regimen, and to increase the salivary pH in the fourth week compared to CON; these changes may have contributed to the greater ruminal pH for TRT reported in those weeks in the companion paper [30]. However, there was no increment in bicarbonate or phosphate due to TRT. These findings indicate that the salivary pH or buffer capacity are also influenced by factors other than salivary major buffers [19,51]. Although the mechanism by which TRT supplementation influences buffer capacity or pH is not clear at the moment, TRT possibly influenced the profile of specific proteins in saliva [58] increasing salivary pH, a topic that deserves further investigation. Another potential explanation to the change in the salivary buffer capacity and pH is the hydration status, as reported in other studies [59], indicating that hydration status affects these salivary variables in other animal species.

## 5. Conclusions

The shift to HG feeding regimens has been perceived as the time with the greatest risk for cattle health because of the major adaptative changes that occur during this period. Our findings show that the negative impacts of an HG challenge are pronounced immediately after the dietary change. However, several effects were worsened by the duration on the HG diet, with host adaptive changes still observed after 3- and 4-weeks following diet change. More specifically, the advanced duration on the HG diet further decreased rumination and total chewing times. In addition, we found that the cows displayed a decreased preference for short feed particles, which might have been a response to modulate ruminal fermentation. The diet change increased the cows’ lying bouts, and these values were maintained throughout the HG feeding challenge, which may reflect the animals’ discomfort. Additionally, we found that the duration of the HG feeding regimen increased salivary pH, which was likely a response to counteract the reduced feed insalivation. Furthermore, the HG rations reduced feed bolus ensalivation in week 4 and salivary phosphate in weeks 1 and 2 on the HG diet. The positive effects of TRT included an increase in salivary pH in week 4 on the HG diet and increased the cows’ preference for fibrous feed particles in week 2 on the HG diet.

Given the effects of diet on animal behavior and salivation dynamics, further research is needed to counteract the negative effects of the HG diets not only during dietary change but also once the cattle have adapted to the HG rations.

## Figures and Tables

**Table 1:** Effect of duration on a high-grain diet on chewing activity of non-lactating Holstein cows not supplemented or receiving a phytogenic feed supplement ^1^.

Item	Week 0		Week 1		Week 2		Week 3		Week 4		SEM ^2^	*p*-Values ^3^		
	CON	TRT	CON	TRT	CON	TRT	CON	TRT	CON	TRT		DUR	TRT	I
Ruminating time, min/d	296	374	353	344	363	301	304	267	273	218	40.8	0.09	0.64	0.22
Eating time, min/d	186	183	156	178	147	157	150	157	146	145	13.3	<0.01	0.63	0.58
Ruminating, chews/min	63.4	63.5	60.7	62.3	61.4	62.3	61.3	63.0	60.9	61.8	0.78	<0.01	0.22	0.53
Ruminating, chews/bolus	47.4	47.0	48.2	49.3	49.4	50.1	50.8	52.7	48.2	50.2	1.52	<0.05	0.45	0.87
Total chewing time, min/d	534	546	490	524	500	464	451	431	426	362	40.2	<0.01	0.67	0.65
Drinking time, min/d *	4.87	5.45	7.51	6.84	8.74	10.28	10.52	8.85	11.63	13.09	1.26	<0.01	0.91	0.66
Drinking gulps, #/day *	68.5	74.5	96.6	97.9	117.8	138.9	148.9	122.0	149.8	188.3	1.25	<0.01	0.85	0.57
Chewing index, chewing time/kg DMI *	63.6	73.7	34.7	32.1	34.6	32.7	32	29.5	32.0 ^x^	27.0 ^y^	1.07	<0.01	0.36	0.07

^1^ CON: A control ration; TRT: inclusion of a phytogenic feed supplement based on L-menthol, thymol, eugenol, mint oil (*Mentha arvensis*), and cloves powder (*Syzygium aromaticum*), (0.04%, DM basis). ^2^ The largest standard error of the mean. ^3^ *p*-values for the effect of duration of high-grain feeding (DUR), phytogenic feed supplement (TRT), and the interaction of duration on high-grain × supplementation (I). * Due to lack of normal distribution, values were first log transformed prior to statistical analysis. ^x, y^ Within corresponding week, means with different superscripts indicate a tendency for a difference (0.05 < *p* ≤ 0.10) between CON and TRT.

**Table 2:** Effect of duration on a high-grain diet on DMI and eating behavior of non-lactating Holstein cows not supplemented or receiving a phytogenic feed supplement ^1^.

	Week 0		Week 1		Week 2		Week 3		Week 4			*p*-Values ^3^		
Item	CON	TRT	CON	TRT	CON	TRT	CON	TRT	CON	TRT	SEM ^2^	DUR	TRT	I
DMI, kg	8.57	7.35	13.5	13.7	13.8	12.6	14.6	13.6	14.1	13.3	0.64	<0.01	0.11	0.69
Valid visits ^4^, #/day	11.9 ^x^	10.3 ^y^	10.9	11.4	10.1	10.5	10.5	10.9	9. 8	9.8	0.71	0.22	0.92	0.43
Visit ^4^ duration, min	10.2	9.9	9.0	9.2	9.4	9.4	8.6	9.5	8.4	9.0	0.49	<0.01	0.59	0.56
Visit ^4^ size, kg DMI	0.57	0.55	1.02	0.97	1.07	0.97	1.34 ^x^	1.06 ^y^	1.04	1.08	0.11	<0.01	0.43	0.51
Meal ^5^ frequency, #/day	6.2 ^x^	5.5 ^y^	4.9	5.4	4.9	5.1	5.1	5.0	4.7	4.7	0.29	<0.01	0.98	0.29
Meal ^5^ duration, min	27.9	28.5	28.1	29.0	28.3	27.3	28.3	27.4	29.7	28.1	0.96	0.45	0.70	0.32
Meal ^5^ size, kg DMI	1.29	1.28	2.82	2.7	2.87 ^x^	2.48 ^y^	2.9	2.69	3.13	2.95	0.15	<0.01	0.28	0.54
Eating rate, g DM/min	46.09	47.09	101.8	94.6	100.2	91.3	103.7	98.8	105	105.6	5.33	<0.01	0.56	0.34

^1^ CON: A control ration; TRT: inclusion of a phytogenic feed supplement based on L-menthol, thymol, eugenol, mint oil (*Mentha arvensis*), and cloves powder (*Syzygium aromaticum*) (0.04%, DM basis). ^2^ The largest standard error of the mean. ^3^ *p*-values for the effect of duration of high-grain feeding (DUR), phytogenic feed supplement (TRT), and the interaction of duration on high-grain × supplementation (I). ^4^ A valid visit was defined when a cow stays in the feeder for at least 4.5 min and consuming at least 200 g of DM. ^5^ A meal was defined as the sum of close visits initiated in less than 29.5 min interval after the end of previous visit. Meal duration calculated as sum of visits + intervals between visits within average meal. ^x, y^ Within corresponding week, means with different superscripts indicate a tendency for a difference (0.05 < *p* ≤ 0.10) between CON and TRT.

**Table 3:** Effect of duration on a high-grain diet on feed sorting behavior of non-lactating Holstein cows not supplemented or receiving a phytogenic feed supplement ^1^.

	Week 1		Week 2		Week 3		Week 4			*p*-Values ^3^		
Particle Fraction ^4^	CON	TRT	CON	TRT	CON	TRT	CON	TRT	SEM ^2^	DUR	TRT	I
Long	92.0	101	81.5 ^b^	107 ^a^	106	102	108	103	7.68	0.36	0.35	0.11
Medium	108	112	116	114	103	107	106	110	3.06	0.01	0.37	0.66
Short	88.0	72.7	73.6	58.3	68.4	67.0	66.4	60.0	8.39	0.08	0.25	0.68
Fine	92.5	71.4	63.8	70.0	81.6	67.3	83.3	65.7	12.29	0.55	0.15	0.57
peNDF ^5^_>8 mm_	106	105	101 ^b^	114 ^a^	104	107	106	106	2.56	0.80	0.07	0.02
peNDF_>1.18 mm_	98.3	99.0	93.3 ^b^	101 ^a^	97.6	100	97.5	96.1	1.51	0.44	0.04	<0.01

^1^ CON: A control ration; TRT: inclusion of a phytogenic feed supplement based on L-menthol, thymol, eugenol, mint oil (*Mentha arvensis*), and cloves powder (*Syzygium aromaticum*) (0.04%, DM basis). ^2^ The largest standard error of the mean. ^3^ *p*-values for the effect of duration of high-grain feeding (DUR), phytogenic feed supplement (TRT), and the interaction of duration on high-grain × supplementation (I). ^4^ Measured according to Kononoff et al. [40]. Values lower than 100 indicate decreased preference (sorting against the corresponding particle fraction); values greater than 100 indicate increased preference (sorting in favor of the corresponding particle fraction). ^5^ Physically effective NDF. ^a, b^ Within corresponding week, means with different superscripts indicate a significant difference (*p* < 0.05) between CON and TRT.

**Table 4:** Effect of duration on a high-grain diet on lying behavior of non-lactating Holstein cows not supplemented or receiving a phytogenic feed supplement ^1^.

	Week 0		Week 1		Week 2		Week 3		Week 4			*p*-Values ^3^		
Item	CON	TRT	CON	TRT	CON	TRT	CON	TRT	CON	TRT	SEM ^2^	DUR	TRT	I
Standing time, h/d	10.8	11.0	10.3	9.2	10.1	10.0	9.7	10.0	10.2	10.6	0.77	0.10	0.93	0.53
Total lying time, h/d	13.2	13.0	13.7	14.8	13.9	14.0	14.3	14.0	13.8	13.5	0.77	0.10	0.93	0.53
Lying time, right side, h/d	6.4	6.4	6.8	6.8	6.4	7.3	5.8 ^y^	6.8 ^x^	6.4	6.4	0.47	0.27	0.38	0.32
Lying time, left side, h/d	6.8	6.8	6.8 ^y^	8.0 ^x^	7.2	6.9	8.2	7.4	7.5	7.2	0.61	0.07	0.95	0.11
Total lying bouts *, #/d	13.6	12.5	16.8	15.2	14.7 ^b^	18.5 ^a^	15.6	15.0	14.3	12.0	1.13	<0.01	0.74	0.14
Lying bouts, right side, #/d	6.3	6.7	9.0	8.7	7.2 ^b^	12.4 ^a^	7.6	8.6	7.2	5.8	1.56	<0.05	0.37	0.10
Lying bouts, left side, #/d	6.2	6.7	8.7	8.5	7.2 ^b^	11.6 ^a^	8.6	8.7	7.4	7.6	1.05	<0.05	0.13	0.06
Rumination standing, min/d ^+^	113	110	74.2	74.7	58.2	57.3	40.1	52.9	48.0	50.6	2.17	0.08	0.82	0.44
Rumination lying right, min/d ^+^	124	98.4	83.1	64.1	65.9	49.4	46.4	44.7	55.0	42.6	2.22	<0.01	0.58	0.97
Rumination lying left, min/d	153 ^y^	266 ^x^	101 ^b^	255 ^a^	127	126	119	74.7	69.1	85.0	3.13	<0.05	0.21	0.11

^1^ CON: A control ration; TRT: inclusion of a phytogenic feed supplement based on L-menthol, thymol, eugenol, mint oil (*Mentha arvensis*), and cloves powder (*Syzygium aromaticum*), (0.04%, DM basis). ^2^ The largest standard error of the mean. ^3^ *p*-values for the effect of duration of high-grain feeding (DUR), phytogenic feed supplement (TRT), and the interaction of duration on high-grain × supplementation (I). ^a, b^ Within corresponding week, means with different superscript indicate a significant difference (*p* < 0.05) between CON and TRT. ^x, y^ Within corresponding week, means with different superscripts indicate a tendency for significant difference (0.05 < *p* ≤ 0.10) between CON and TRT. * Due to lack of normal distribution, values were first log-transformed prior to statistical analysis. ^+^ Due to lack of normal distribution, values were first root square-transformed prior to statistical analysis.

**Table 5:** Effect of a dietary shift from only forage to a high-grain diet on feed bolus size and salivation of non-lactating Holstein cows not supplemented or receiving a phytogenic feed supplement ^1^.

	Week 0		Week 4			*p*-Values ^3^		
Item	CON	TRT	CON	TRT	SEM ^2^	DI	TRT	I
Feed bolus size (as is), g	239	245	302	283	22.6	<0.05	0.76	0.55
Feed bolus size (DM), g	31.6	29.8	70.5	64.8	5.7	<0.01	0.49	0.72
Saliva in bolus, g	139	150	143	146	10.9	0.97	0.51	0.71
Feed ensalivation, g saliva/g feed	5.31	6.32	2.74	3.00	0.48	<0.01	0.13	0.59
Saliva flow, g/min	69.7	75.0	71.8	73.3	4.49	0.97	0.51	0.71

^1^ CON: A control ration; TRT: inclusion of a phytogenic feed supplement based on L-menthol, thymol, eugenol, mint oil (*Mentha arvensis*), and cloves powder (*Syzygium aromaticum*) (0.04%, DM basis). ^2^ The largest standard error of the mean. ^3^ *p*-values for the effect of diet (DI), phytogenic feed supplement (TRT), and the interaction of diet × supplementation (I).

**Table 6:** Effect of duration on a high-grain diet on salivary physico-chemical properties of non-lactating Holstein cows not supplemented or receiving a phytogenic feed supplement ^1^.

	Week 0		Week 1		Week 2		Week 3		Week 4			*p*-Values ^3^		
Item	CON	TRT	CON	TRT	CON	TRT	CON	TRT	CON	TRT	SEM ^2^	DUR	TRT	I
Salivary pH	8.86	8.88	8.96	8.87	8.94	8.98	9.02	9.04	8.86 ^b^	9.02 ^a^	0.05	<0.05	0.59	0.11
Buffer capacity, decamol HCl/L/ΔpH	0.013	0.014	0.014	0.013	0.013	0.014	0.014 ^y^	0.015 ^x^	0.014	0.014	0.001	0.64	0.18	0.26
Bicarbonate, mM	67.7	69.6	73.6	68.5	67.8	70.0	73.1	71.8	73.4	74.1	3.68	0.20	0.93	0.64
Phosphate, mM	11.7	12.2	9.95	10.8	10.4	10.9	13.1	13.8	12.7	12.2	0.89	<0.01	0.64	0.88
Mucin, mg/mL	1.75	1.30	1.30	1.48	1.41	1.83	1.19	1.23	1.10	1.16	0.25	0.15	0.84	0.40
Lysozyme activity, U/mL/min *	24.9	26.4	45.1	34.6	39.6	46.2	42.8	49.7	48.1	44.9	1.20	<0.01	0.96	0.63
Total protein, µg/mL	445	442	404	390	529	486	405	381	452	404	55.0	0.56	0.13	0.99

^1^ CON: A control ration; TRT: inclusion of a phytogenic feed supplement based on L-menthol, thymol, eugenol, mint oil (*Mentha arvensis*), and cloves powder (*Syzygium aromaticum*) (0.04%, DM basis). ^2^ The largest standard error of the mean. ^3^ *p*-values for the effect of duration of high-grain feeding (DUR), phytogenic feed supplement (TRT), and the interaction of duration on high-grain × supplementation (I). * Due to lack of normal distribution, values were first log transformed prior to statistical analysis. ^a, b^ Within corresponding week, means with different superscripts indicate a significant difference (*p* < 0.05) between CON and TRT. ^x, y^ Within corresponding week, means with different superscripts indicate a tendency for a difference (0.05 < *p* ≤ 0.10) between CON and TRT.

## Data Availability

Data are contained within this article or in the Supplementary Material.

## Supplementary Materials

The following supporting information can be downloaded at: https://www.mdpi.com/article/10.3390/ani12152001/s1, Table S1: Ingredients, chemical composition, particle size fractions, and physically effective fiber of the diets fed to cows during the study.
