# Supplementary material for: Effect of Duration of High-Grain Feeding on Chewing, Feeding Behavior, and Salivary Composition in Cows with or without a Phytogenic Feed Supplement
Source: Animals (Basel). 2022 Aug 8;12(15):2001. doi: 10.3390/ani12152001 (PMC9367443; doi:10.3390/ani12152001)
Supplement: Supplementary file 1 [file animals-12-02001-s001.zip › animals-1805954-supplementary.pdf]

Supplementary material

Table S1. Ingredients, chemical composition, particle size fractions, and physically effective fiber of the diets fed to cows during the study.

Item	Diet and treatment, % DM (unless otherwise stated)		
	Forage diet*	High-grain diet CON	High-grain diet TRT
Ingredients			
Grass silage	75	26.25	26.25
Corn silage	15	8.75	8.75
Grass hay	10	0	0
Control concentrate[1]	0	65	0
Treatment concentrate[2]	0	0	65
TMR chemical composition			
DM, % as fresh	32.4	45.1	44.0
Crude protein, %	17.2	19.6	19.3
Neutral detergent fiber, %	50.4	30.2	31.6
Acid detergent fiber), %	36.6	19.9	20.0
Starch, %	4.2	28.9	28.0
Ether extract, %	2.9	3.2	3.2
Non-fiber carbohydrates, %	18.4	39.5	39.0
Ash, %	11.0	6.8	6.7
Particle fraction (% retained)[3]			
Long	86.7	27.8	29.2
Medium	5.54	29.3	29.7
Short	7.30	20.3	18.8
Fine	0.50	1.4	1.1
Physical effectiveness factor	0.92	0.6	0.6
Physically effective NDF>8 mm	47.5	17.3	18.6

[1]CON: The control concentrate mixture contained: barley grain (30.22%), triticale grain (18.1%), bakery by-product (23.08%), rapeseed meal (24.0%), molasses (3.0%), mineral-vitamin premix for dairy cattle (0.8%), limestone (0.5%), and salt (0.3%).

[2]TRT: The treatment concentrate mixture contained: barley grain (30.22%), triticale grain (18.04%), bakery by-product (23.08%), rapeseed meal (24.0%), molasses (3.0%), mineral-vitamin premix for dairy cattle (0.8%), limestone (0.5%), salt (0.03%). Formulated to provide 0.04% in the TMR of a phytogenic feed supplement based on L-menthol, thymol, eugenol, mint oil (*Mentha arvensis*) and cloves powder (*Syzygium aromaticum*), (TRT, Digestarom®, DSM Austria GmbH).

*This diet was common for both CON and TRT cows; during this week of baseline forage feeding, the mineral and vitamin premix without (CON) or with the phytogenic feed supplement (TRT) was introduced into the rumen through the ruminal cannula before the morning feeding to corresponding cows.

[3]Particle fractions determined with the Penn State Particle Separator with a 19-mm screen (long), 8-mm screen (medium), 1.18-mm screen (short), and a pan (fine), (Kononoff et al. 2003).
